# Gemini 1.5 Flash provides the most reliable content while ChatGPT‐4o offers the highest readability for patient education on meniscal tears

**DOI:** 10.1002/ksa.70247

**Published:** 2025-12-26

**Authors:** Başar Burak Çakmur, Ali Can Koluman, Mehmet Utku Çiftçi, Ebru Aloğlu Çiftçi, Nezih Ziroğlu

**Affiliations:** ^1^ Department of Orthopedics and Traumatology Beylikduzu State Hospital Istanbul Turkey; ^2^ Department of Orthopedics and Traumatology Bakirkoy Dr. Sadi Konuk Training and Research Istanbul Turkey; ^3^ Department of Orthopedics and Traumatology Sultan Abdulhamid Han Training and Research Hospital Istanbul Turkey; ^4^ Faculty of Health Sciences, Department of Physiotherapy and Rehabilitation Istanbul Okan University Istanbul Turkey; ^5^ Institute of Graduate Education, Department of Physiotherapy and Rehabilitation Istinye University Istanbul Turkey; ^6^ Vocational School of Health Services, Department of Orthopedic Prosthetics and Orthotics Acıbadem Mehmet Ali Aydınlar University Istanbul Turkey; ^7^ Department of Orthopaedics and Traumatology Acibadem University Atakent Hospital Istanbul Turkey

**Keywords:** ChatGPT, DeepSeek, Gemini, large language models, meniscal tear, patient education

## Abstract

**Purpose:**

The aim of this study was to comparatively evaluate the responses generated by three advanced artificial intelligence (AI) models, ChatGPT‐4o (OpenAI), Gemini 1.5 Flash (Google) and DeepSeek‐V3, to frequently asked patient questions about meniscal tears in terms of reliability, usefulness, quality, and readability.

**Methods:**

Responses from three AI chatbots, ChatGPT‐4o (OpenAI), Gemini 1.5 Flash (Google) and DeepSeek‐V3 (DeepSeek AI), were evaluated for 20 common patient questions regarding meniscal tears. Three orthopaedic specialists independently scored reliability and usefulness on 7‐point Likert scales and overall response quality using the 5‐point Global Quality Scale. Readability was analysed with six established indices. Inter‐rater agreement was examined with intraclass correlation coefficients (ICCs) and Fleiss’ Kappa, while between‐model differences were tested using Kruskal–Wallis and ANOVA with Bonferroni adjustment.

**Results:**

Gemini 1.5 Flash achieved the highest reliability, significantly outperforming both GPT‐4o and DeepSeek‐V3 (*p* = 0.001). While usefulness scores were broadly similar, Gemini was superior to DeepSeek‐V3 (*p* = 0.045). Global Quality Scale scores did not differ significantly among models. In contrast, GPT‐4o consistently provided the most readable content (*p* < 0.001). Inter‐rater reliability was excellent across all evaluation domains (ICC > 0.9).

**Conclusion:**

All three AI models generated high‐quality educational content regarding meniscal tears. Gemini 1.5 Flash demonstrated the highest reliability and usefulness, while GPT‐4o provided significantly more readable responses. These findings highlight the trade‐off between reliability and readability in AI‐generated patient education materials and emphasise the importance of physician oversight to ensure safe, evidence‐based integration of these tools into clinical practice.

**Level of Evidence:**

Level V, observation‐based, expert opinion‐based, or in vitro/artificial intelligence model evaluation.

**Abbreviations**
AI: artificial intelligence
ANOVA: analysis of variance
ARI: Automated Readability Index
CLI: Coleman–Liau Index
DeepSeek‐V3: (DeepSeek AI model)
FKGL: Flesch‐Kincaid Grade Level
FRE: Flesch Reading Ease
Gemini 1.5 Flash: (Google model)
GFI: Gunning Fog Index
GPT‐4o: Generative Pre‐trained Transformer 4 Omni (OpenAI model)
GQS: Global Quality Scale
ICC/ICCs: intraclass correlation coefficient(s)
IQR: interquartile range
LLM/LLMs: large language model(s)
MRI: magnetic resonance imaging
PRP: platelet‐rich plasma
SD: standard deviation
SMOG: Simple Measure of Gobbledygook
η²: eta squared

## INTRODUCTION

The integration of artificial intelligence (AI), particularly large language models (LLMs), is rapidly transforming medical applications, including diagnosis, treatment planning and patient education [6, 10, 23, 28]. However, the reliability, accuracy and comprehensibility of AI‐generated content remain a major concern [1, 5]. Meniscal tears are highly common, representing a frequent subject of patient information‐seeking where access to comprehensible information is critical [14].

Recent evaluations of chatbots for orthopaedic education show heterogeneity; some studies report accurate content that lacks readability [5], while others comparing new models on topics like ACL surgery show performance variability, with some excelling in comprehensiveness and others in clarity [11, 20]. The emergence of new models like DeepSeek further complicates this landscape [16].

While AI's potential in orthopaedics is recognised [13, 15], a significant gap exists. No study has directly compared the newest generation of flagship models (GPT‐4o, Gemini 1.5 Flash and DeepSeek‐V3) on the dual metrics of reliability and readability for a common orthopaedic condition. This is clinically relevant because clinicians and patients increasingly encounter these tools, yet lack data on which model provides the safest (most reliable) versus the most understandable (most readable) information. Understanding this trade‐off is essential for the safe, evidence‐based integration of AI into clinical practice.

Therefore, the aim of this study was to comparatively evaluate these three models. It was hypothesised that no single AI model would be superior across all metrics. Specifically, it was predicted that the models would exhibit a critical trade‐off: some models would produce content with higher factual reliability and usefulness, while others would excel in linguistic simplicity and readability.

## MATERIALS AND METHODS

### Study design

This study evaluated the responses of GPT‐4o (OpenAI), Gemini 1.5 Flash (Google), and DeepSeek‐V3 (DeepSeek AI) to frequently asked patient questions about meniscal tears. All AI‐generated responses were collected between June and July 2025. During this period, GPT‐4o was accessed via ChatGPT (OpenAI), Gemini 1.5 Flash via Google AI Studio, and DeepSeek‐V3 through its official web interface. Each model version was used consistently throughout the data collection period to ensure reproducibility. In the first stage, a multi‐platform AI querying approach was used. Each AI model was prompted with: ‘What are the most frequently asked patient questions about meniscal tears?’ The lists of questions generated were compiled and cross‐referenced. A consensus list of overlapping questions was created, which was then refined with Google Trends data using relevant keywords (e.g., ‘meniscal tear’, ‘meniscus surgery’, ‘meniscus pain’). Through this process, 20 frequently asked patient questions were identified (Table 1). These were reviewed and validated for content by three orthopaedic specialists and classified into three categories: diagnosis/symptoms (*n* = 7), treatment/intervention (n = 8), and lifestyle/activity (*n* = 5).The final number of 20 questions was not predetermined. After completing all cross‐referencing and validation steps across AI‐generated lists and Google Trends data, a total of 20 unique and representative patient questions remained. This number emerged naturally from the data synthesis process rather than being selected a priori, and it was considered sufficient to represent the most frequently asked questions about meniscal tears.

**Table 1:** Frequently asked patient questions regarding meniscal tears.

Domain	Questions
Diagnosis and symptoms	What is a meniscal tear? How is a meniscal tear diagnosed? What are the symptoms of a meniscal tear? Can a meniscal tear be definitively diagnosed with magnetic resonance imaging (MRI)? What causes the knee to lock or catch? Is knee pain always indicative of a meniscal tear? 7. Can a meniscal tear heal spontaneously?
Treatment and interventions	8. Can a meniscal tear heal without surgical intervention? 9. Is surgery always necessary for a meniscal tear? 10. How is meniscal surgery performed? 11. What is arthroscopy, and is it a painful procedure? 12. Is physical therapy effective in treating meniscal tears? 13. Are platelet‐rich plasma (PRP) or stem cell therapies effective for meniscal injuries? 14. How long does recovery take after meniscal surgery? 15. Will I be able to use my knee as before after meniscal surgery?
Resumption of activity and long‐term prognosis	16. Can individuals with a meniscal tear participate in sports activities? 17. When can I return to sports after a meniscal injury? 18. Can the meniscus be damaged during exercise? 19. Can meniscal tears recur? 20. Can a meniscal tear lead to knee arthritis or the need for a prosthesis?

In the second stage, the 20 questions were posed separately to each AI model by a volunteer not involved in the study. All responses were saved in Microsoft Word (version 16.98, Microsoft Corp., Redmond, WA, USA). To minimise bias, responses were anonymized and coded prior to analysis.

### Evaluation of responses

The evaluations were conducted by three independent, board‐certified orthopaedic surgeons, each with over five years of clinical experience in musculoskeletal disorders. Reliability was assessed on a 7‐point Likert scale (1 = completely unreliable, 7 = completely reliable) based on consistency with current literature and accuracy of medical information. Usefulness was assessed on a 7‐point Likert scale considering clarity, comprehensiveness, and practical applicability [9, 24].

Response quality was measured using the previously validated 5‐point Global Quality Scale (GQS), where 1–2 indicated poor/inadequate content, 3 indicated limited but essential information, and 4–5 indicated high‐quality, comprehensive, and scientifically reliable responses [9, 21].

Readability was assessed using multiple indices: Flesch Reading Ease (FRE), Flesch‐Kincaid Grade Level (FKGL), Gunning Fog Index (GFI), Coleman‐Liau Index (CLI), SMOG Index, and Automated Readability Index (ARI). Scores were calculated using UK‐based online readability tools (e.g., https://readable.com). Higher FRE scores indicated easier readability, while grade‐level indices reflected approximate education level required for comprehension. As this study did not involve human or animal subjects, institutional ethics approval was not required.

### Statistical analysis

All analyses were conducted using IBM SPSS Statistics 27.0 (IBM Corp., Armonk, NY, USA). Descriptive statistics were presented as mean ± standard deviation (SD), median, and interquartile range (IQR). Normality was assessed using the Shapiro–Wilk test. Inter‐rater agreement was evaluated using Fleiss’ Kappa and Intraclass Correlation Coefficient (ICC). Fleiss’ Kappa was categorised as moderate (0.2–0.4), fair (0.4–0.6), strong (0.6–0.8), and almost perfect (0.8–1.0) [17, 19]. ICC was calculated using a two‐way random‐effects model with absolute agreement, interpreted as poor ( < 0.2), moderate (0.2–0.4), acceptable (0.4–0.6), good (0.6–0.8), and excellent (0.8–1.0) reliability [19].

Between‐group comparisons were performed using Kruskal‐Wallis test with Bonferroni‐Dunn adjusted Mann–Whitney U tests for post‐hoc analysis. For normally distributed data, one‐way ANOVA with Bonferroni correction was used. Effect sizes were reported using eta squared (η²). Statistical significance was set at *p* < 0.05.

## RESULTS

### Inter‐rater agreement

Excellent inter‐rater agreement was observed across all evaluation domains. Reliability scores demonstrated excellent agreement (ICC > 0.9). Fleiss’ Kappa coefficients also indicated strong to almost perfect agreement for reliability, usefulness, and GQS assessments. All inter‐rater agreement values were statistically significant (*p* < 0.001), and full details are presented in Table 2.

**Table 2:** Inter‐rater agreement of AI‐generated responses across models and evaluation criteria (ICC and Fleiss' kappa) *(all ICC and Fleiss’ Kappa values were statistically significant [p* < *0.001])*.

Model	Criterion	ICC (95% CI)	Interpretation (ICC)	Fleiss’ kappa	Interpretation (kappa)
ChatGPT‐4o	Reliability	0.905 (0.802–0.959)	Excellent agreement	0.630	Strong agreement
	Usefulness	0.968 (0.932–0.986)	Excellent agreement	0.878	Almost perfect
	GQS	0.923 (0.840–0.967)	Excellent agreement	0.792	Strong agreement
Gemini 1.5 Flash	Reliability	0.861 (0.711–0.940)	Excellent agreement	0.659	Strong agreement
	Usefulness	0.959 (0.914–0.982)	Excellent agreement	0.880	Almost perfect
	GQS	0.933 (0.859–0.971)	Excellent agreement	0.814	Almost perfect
DeepSeek‐V3	Reliability	0.957 (0.909–0.982)	Excellent agreement	0.745	Strong agreement
	Usefulness	0.976 (0.950–0.990)	Excellent agreement	0.890	Almost perfect
	GQS	0.990 (0.978–0.996)	Excellent agreement	0.946	Almost perfect

### Reliability

A statistically significant difference was detected in the reliability of responses to diagnosis‐ and symptom‐related questions (*p* = 0.006). Gemini 1.5 Flash was significantly more reliable than DeepSeek‐V3 (padj = 0.024). For treatment‐ and intervention‐related questions, significant differences were also observed (*p* = 0.004). Gemini 1.5 Flash demonstrated higher reliability compared with both GPT‐4o (padj = 0.009) and DeepSeek‐V3 (padj = 0.015).

**Figure 1:**
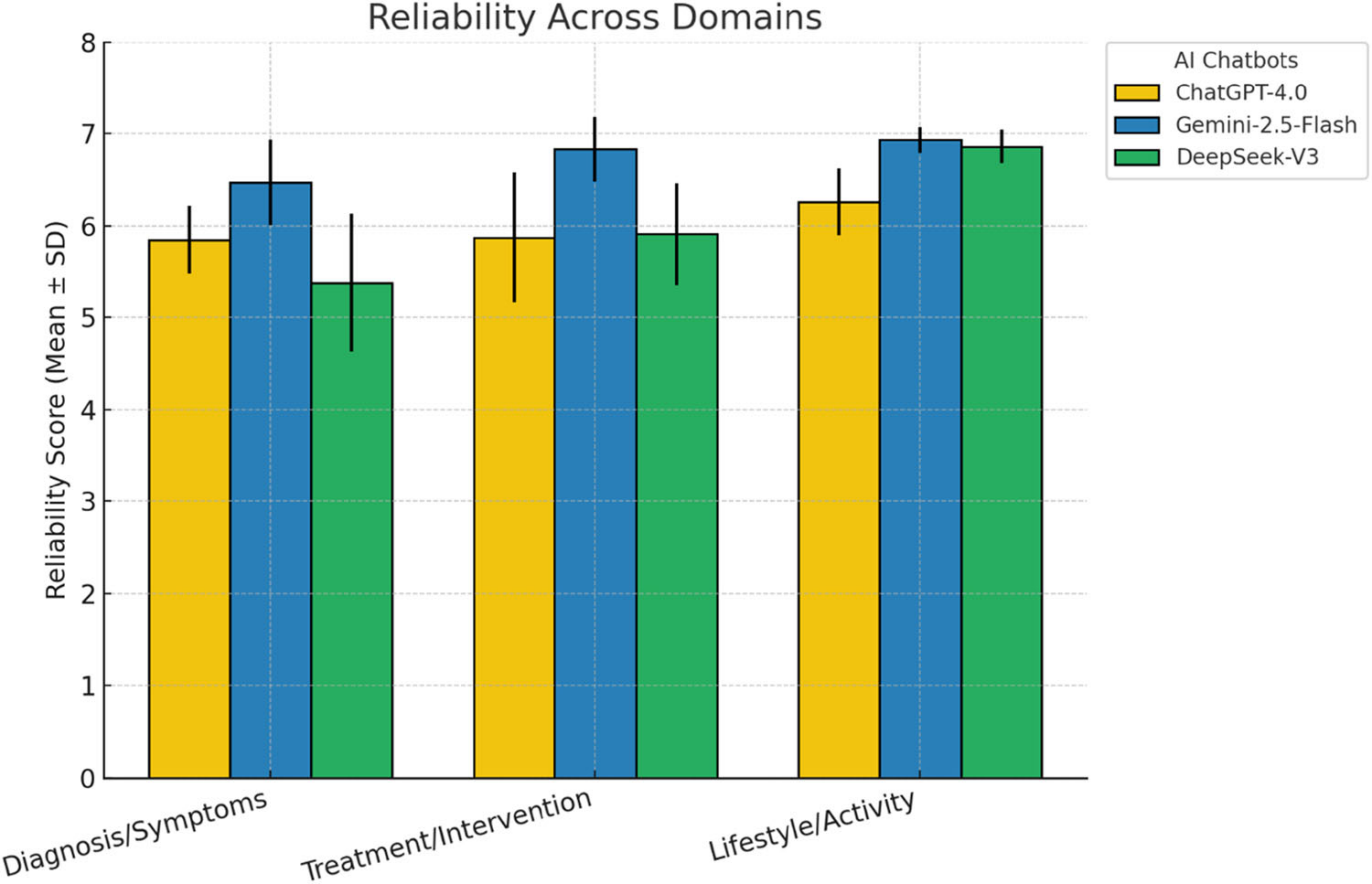
Mean ± SD reliability scores of ChatGPT‐4o, Gemini 1.5 Flash, and DeepSeek‐V3 across clinical domains.

In lifestyle‐ and activity‐related questions, Gemini 1.5 Flash was significantly more reliable than GPT‐4o (padj = 0.003), while no differences were observed between GPT‐4o and DeepSeek‐V3 (n.s.) or between DeepSeek‐V3 and Gemini 1.5 Flash (n.s.). When all questions were analysed collectively, Gemini 1.5 Flash achieved significantly higher overall reliability (*p* = 0.001; Figure 1).

### Usefulness and Global Quality Scale (GQS)

**Figure 2:**
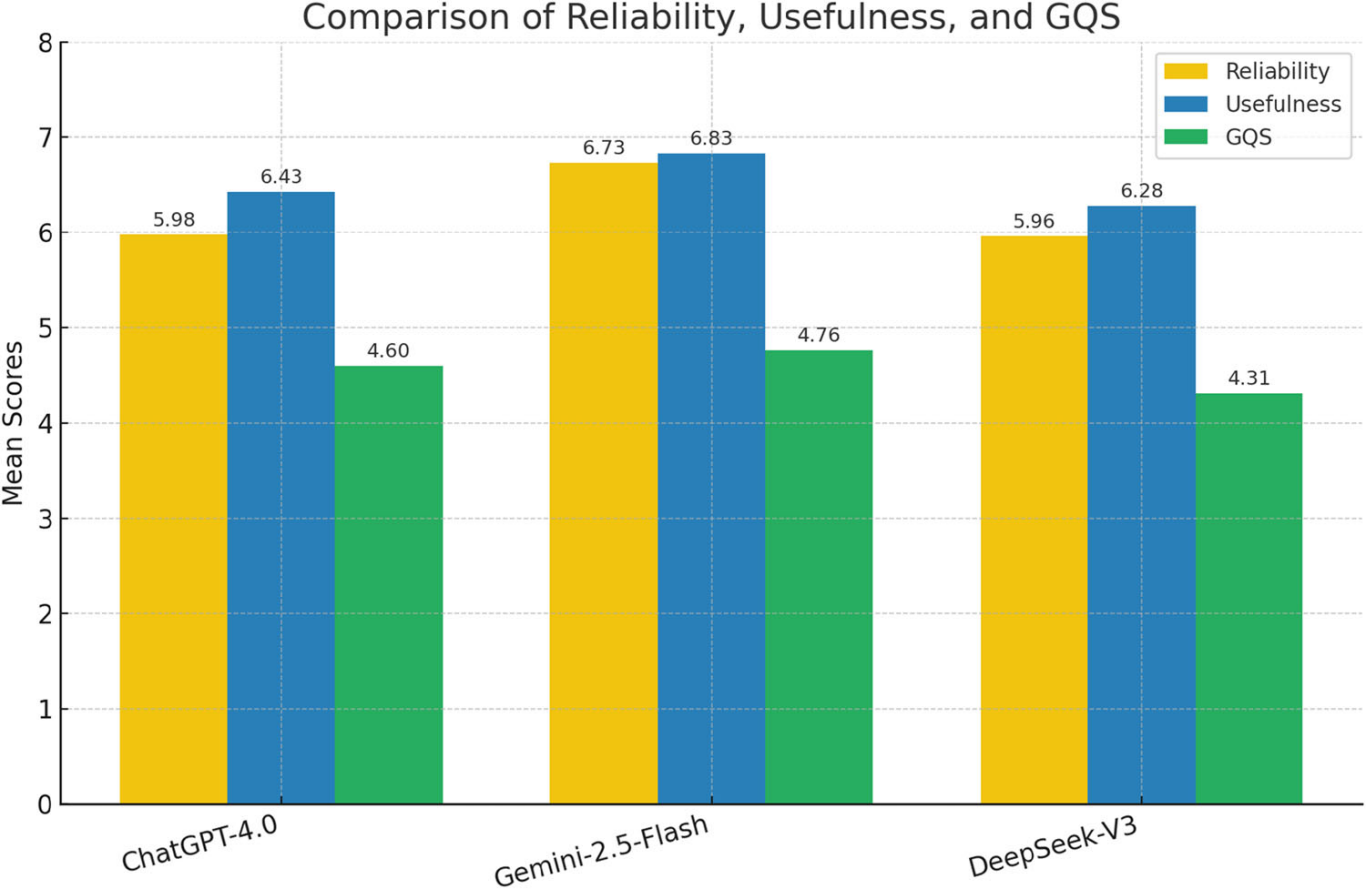
Comparison of mean reliability, usefulness, and GQS scores across AI models Global Quality Scale (GQS).

Usefulness evaluations by question category revealed no significant differences among the models (Table 3). However, when all questions were combined, a significant difference was detected (*p* = 0.014), with Gemini 1.5 Flash scoring significantly higher than DeepSeek‐V3 (padj = 0.045). In contrast, mean GQS scores did not reach statistical significance among the models (n.s.) (Table 3). A comparative overview is shown in Figure 2.

**Table 3:** Reliability, usefulness and quality (GQS) scores of AI‐generated responses on meniscus tears.

Evaluation criteria	Domain/category	ChatGPT‐4o (mean ± SD, median [IQR])	Gemini 1.5 Flash (mean ± SD, median [IQR])	DeepSeek‐V3 (mean ± SD, median [IQR])	Overall *p*	Significant pairwise differences
Reliability	Diagnosis and symptoms	5.85 ± 0.37, 6 (0)	6.47 ± 0.46, 6.66 (1)	5.38 ± 0.75, 5.66 (1)	0.006	Gemini > DeepSeek (*p* = 0.024)
	Treatment and intervention	5.87 ± 0.71, 6 (1.25)	6.83 ± 0.35, 7 (0.25)	5.91 ± 0.55, 6 (0.33)	0.004	Gemini > ChatGPT (*p* = 0.009); Gemini > DeepSeek (*p* = 0.015)
	Lifestyle and activity	6.26 ± 0.36, 6 (0.67)	6.93 ± 0.14, 7 (0.17)	6.86 ± 0.18, 7 (0.33)	0.013	Gemini > ChatGPT (*p* = 0.003)
	Overall total	5.98 ± 0.54, 6 (0.25)	6.73 ± 0.39, 7 (0.33)	5.96 ± 0.80, 6 (1)	0.001	Gemini > ChatGPT (*p* = 0.003); Gemini > DeepSeek (*p* = 0.003)
Usefulness	Diagnosis and symptoms	6.28 ± 0.48, 6 (1)	5.95 ± 0.65, 6 (1)	5.95 ± 0.65, 4.66 (1)	0.113	– (ns)
	Treatment and intervention	6.37 ± 0.74, 6.5 (1)	6.87 ± 0.35, 7 (1)	6.12 ± 0.64, 6 (0.75)	0.051	– (ns)
	Lifestyle and activity	6.80 ± 0.29, 7 (0.5)	7.00 ± 0.00, 7 (0)	7.00 ± 0.00, 7 (0)	0.117	– (ns)
	Overall total	6.43 ± 0.59, 6.33 (1)	6.83 ± 0.36, 7 (0)	6.28 ± 0.68, 6 (1)	0.014	Gemini > DeepSeek (*p* = 0.045)
GQS	Diagnosis and symptoms	4.47 ± 0.46, 4.66 (1)	4.61 ± 0.44, 4.66 (1)	4.04 ± 0.59, 4 (0.33)	0.175	– (ns)
	Treatment and intervention	4.62 ± 0.51, 5 (1)	4.75 ± 0.46, 5 (1)	4.12 ± 0.83, 4 (1.75)	0.196	– (ns)
	Lifestyle and activity	4.73 ± 0.43, 5 (0.67)	5.00 ± 0.00, 5 (0)	5.00 ± 0.00, 5 (0)	0.117	– (ns)
	Overall total	4.60 ± 0.46, 4.83 (1)	4.76 ± 0.40, 5 (0.33)	4.31 ± 0.72, 4.16 (1)	0.118	– (ns)

Abbreviation: ns, no significant difference.

### Readability analysis

Table 4 compares the readability of the responses from the AI models, where statistically significant differences were found in all indices (*p* < 0.001). ChatGPT‐4o produced the most readable content with the highest FRE and CLI scores (*p* < 0.001). Deepseek‐V3 provided the simplest content in GFI and ARI results; ChatGPT‐4o and Deepseek‐V3 produced more readable text compared to Gemini1.5Flash (*p* < 0.001). No difference was found between ChatGPT‐4o and DeepSeek‐V3 according to the SMOG and FKGL indices (n.s.) (Table 4).

**Table 4:** Comparison of readability scores among AI models based on different readability metrics.

Readability metrics	AI models	Mean ± SD	Median (IQR)	η²	Test statistic	*p* (*p* adj)
FRE	ChatGPT‐4o	28.47 ± 8.20	29.60 (8.75)	0.589	40.796	<0.001* (<0.001b, 0.050c, < 0.001 d)
	Gemini 1.5 Flash	5.79 ± 7.73	7.00 (11.63)			
	Deepseek V3	22.07 ± 8.59	24.10 (13.70)			
FKGL	ChatGPT‐4o	11.60 ± 2.52	10.98 (2.04)	0.517	31.482	<0.001** (<0.001b, 0.099c, < 0.001 d)
	Gemini 1.5 Flash	16.47 ± 2.19	15.86 (2.00)			
	Deepseek V3	12.80 ± 1.78	12.45 (2.77)			
GFI	ChatGPT‐4o	14.92 ± 2.12	15.00 (2.22)	0.576	34.810	<0.001** (<0.001b, 0.015c, < 0.001 d)
	Gemini 1.5 Flash	18.50 ± 1.76	18.20 (1.78)			
	Deepseek V3	12.68 ± 2.45	11.60 (3.75			
CLI	ChatGPT‐4o	12.35 ± 1.42	12.46 (2.49)	0.591	41.131	<0.001* (<0.001b, 0.012c, < 0.001 d)
	Gemini 1.5 Flash	17.08 ± 1.60	16.92 (2.53)			
	Deepseek V3	13.93 ± 1.97	13.70 (2.75)			
SMOG	ChatGPT‐4o	9.57 ± 1.91	9.29 (0.85)	0.390	24.256	<0.001** (<0.001b, 1.251c, < 0.001 d)
	Gemini 1.5 Flash	12.85 ± 1.03	12.89 (1.26)			
	Deepseek V3	10.43 ± 2.21	9.40 (2.75)			
ARI	ChatGPT‐4o	12.89 ± 1.53	12.77 (2.05)	0.662	39.747	<0.001** (<0.001b, < 0.001c, < 0.001 d)
	Gemini 1.5 Flash	17.47 ± 2.62	17.94 (2.80)			
	Deepseek V3	9.10 ± 2.93	9.55 (4.79)			

## DISCUSSION

The most important finding of the present study was the identification of a clear trade‐off between reliability and readability among the evaluated AI models. Gemini 1.5 Flash provided the most reliable responses, whereas ChatGPT‐4o consistently produced more readable content, with DeepSeek‐V3 showing intermediate performance. Overall response quality was similar among the three models.

The findings of this study align with a growing body of research demonstrating that AI chatbots can provide clinically acceptable content on orthopaedic conditions [10, 28]. For instance, Winden et al. reported satisfactory answers to common questions on meniscus surgery [25], and similar positive findings have been reported for topics like hip arthroscopy [4], total ankle arthroplasty [2], and even spine surgery research [12], and rotator cuff tears [18]. The absence of significant differences in GQS scores across the tested models is consistent with these observations [14], suggesting that current LLMs have reached a baseline threshold of medical acceptability for enhancing patient information [27] and supporting auxiliary care [26].

A consistent theme across comparative studies is model‐dependent heterogeneity rather than a single ‘best’ chatbot. In this analysis, Gemini 1.5 Flash delivered the highest overall reliability. This contrasts with recent work on ACL education by Gültekin et al., which found ChatGPT‐4o excelled in comprehensiveness while DeepSeek R1 led in readability [11]. Similarly, marked variability among Claude, GPT, and Gemini has been noted in perioperative counselling for superior capsular reconstruction [20]. The introduction of new models like DeepSeek further complicates this landscape [16]. Likewise, prospective diagnostic assessments have demonstrated that performance fluctuates by task, dataset, and prompt structure [22]. Taken together, these findings support a topic‐ and use‐case–contingent approach to deployment: model choice should be aligned with the primary objective, clinical domain, and the evaluation rubric [11, 20, 22].

Readability emerged as a decisive factor in determining the real‐world utility of AI‐generated content. Prior analyses of online patient resources have often shown linguistic complexity that exceeds recommended comprehension levels. In contrast, the present study demonstrated that GPT‐4o consistently outperformed Gemini and DeepSeek in readability indices, highlighting its strength in simplifying complex medical information for a broad audience. However, this accessibility was accompanied by a modest trade‐off in reliability compared with Gemini. A similar paradox has been described in ankle sprain–related queries, where more comprehensible outputs were not necessarily the most accurate [7]. These findings reinforce that readability alone cannot guarantee safe or evidence‐based patient education, and emphasise the necessity of clinician oversight to contextualise AI outputs.

The rapid integration of AI into orthopaedics has sparked debate about whether such systems could eventually replace physicians [8] and who should retain ultimate autonomy [3]. The present findings, in line with recent evidence, suggest that this concern is overstated. While LLMs can generate medically acceptable responses, they remain incapable of incorporating individual patient factors such as comorbidities, treatment preferences, or psychosocial context. As emphasised by Al Ramlawi et al., AI‐generated material must undergo rigorous appraisal for accuracy, clarity, and safety before clinical use [1]. Thus, these technologies should be viewed not as substitutes for clinical expertise, but as adjunctive tools that can enhance patient education and support shared decision‐making when used under physician supervision.

The central clinical implication of this work is the tension between reliability and readability in AI‐mediated patient education. Gemini 1.5 Flash delivered the most reliable responses, whereas GPT‐4o offered the greatest accessibility; DeepSeek‐V3 performed intermediately. This divergence highlights a critical point: accurate yet complex information may not be readily understood, while simplified content may omit nuance or precision. Physicians therefore remain indispensable as gatekeepers, ensuring that AI‐generated material is translated into safe, individualised guidance.

Strengths of this study include the direct head‐to‐head comparison of three state‐of‐the‐art models, the use of a multidimensional evaluation framework, and excellent inter‐rater agreement. However, several limitations must be acknowledged in detail. First, the study was limited to English‐language outputs, and the performance of these models may differ significantly in other languages. Second, the limited number of questions (*n* = 20), while carefully curated, may not capture the full spectrum of patient queries; a larger or different set might yield different performance trade‐offs. Third, LLMs are not static; they undergo constant updates, meaning these specific results may change as models evolve. Finally, and most importantly, this study did not include patient‐reported outcomes, such as actual comprehension, user satisfaction, or impact on decision‐making, which are critical for assessing real‐world utility.

The findings of this study have direct relevance for daily clinical practice. They confirm that while AI models are powerful tools for generating patient information, they are not interchangeable. The clear ‘reliability vs. readability’ gap underscores that physicians must act as essential gatekeepers. In practice, a clinician might use a highly readable model like ChatGPT‐4o to generate a simple draft for a patient handout, but must then rigorously cross‐check and edit that draft against evidence‐based sources (or a more reliable model like Gemini) to ensure factual accuracy before giving it to a patient. This study provides the evidence needed to guide this ‘human‐in‐the‐loop’ oversight, ensuring that AI is used as a supportive adjunct, not a replacement for clinical expertise, to deliver safe patient care.

## CONCLUSION

In summary, all three evaluated LLMs provided medically acceptable information on meniscal tears, but their strengths diverged: Gemini 1.5 Flash proved most reliable, while GPT‐4o was most readable. These findings underscore that accuracy and readability do not always align, confirming the essential role of physicians in contextualising AI‐generated information to ensure safe patient care.

## AUTHOR CONTRIBUTIONS

**Başar Burak Çakmur, Ali Can Koluman** and **Mehmet Utku Çiftçi**: conceptualisation, methodology, data curation, formal analysis, writing – review and editing. **Ebru Aloğlu Çiftçi** and **Nezih Ziroğlu**: conceptualisation, writing – original draft preparation, supervision. **Nezih Ziroğlu**: correspondence and project administration.

## CONFLICT OF INTEREST STATEMENT

The authors declare no conflicts of interest.

## ETHICS STATEMENT

As this study exclusively utilised publicly available data generated by artificial intelligence models and did not involve human participants, human material, or animals, the requirement for formal ethical approval was waived by the Institutional Review Board. This study did not involve human participants or patient data; it evaluated publicly available artificial intelligence model outputs.

## Data Availability

The data that support the findings of this study are available from the corresponding author upon reasonable request.
